# Application of Sterile Insect Technique (SIT) for *Aedes albopictus* (Skuse, 1895) in Sri Lanka: Dose optimization, mating competitiveness and release ratios

**DOI:** 10.1371/journal.pone.0331452

**Published:** 2025-09-04

**Authors:** Jeevanie Harishchandra, Wimaladharma Abeyewickreme, Risintha Premaratne, Menaka Hapugoda

**Affiliations:** 1 Anti-Malaria Campaign, Ministry of Health, Colombo, Sri Lanka; 2 Molecular Medicine Unit, Faculty of Medicine, University of Kelaniya, Ragama, Sri Lanka; 3 Department of Parasitology, Faculty of Medicine, University of Kelaniya, Ragama, Sri Lanka; 4 Department of Communicable Diseases, World Health Organization, Regional Office for South-East Asia, New Delhi, India; Al-Azhar University, EGYPT

## Abstract

**Background:**

Sri Lanka has experienced severe dengue epidemics in recent years, despite the extensive vector control measures taken. Therefore, it is necessary to find sustainable vector control strategies against dengue. Novel vector control tools need to be tested for the feasibility of applying them against local vectors. Sterile Insect Technique (SIT) is an increasingly popular vector control technique which has been adopted by many countries to suppress insect pest populations and is being tested for dengue vectors.

**Methods:**

In this study, SIT was developed for *Aedes albopictus* (Skuse, 1895), one of the 2 dengue vectors present in Sri Lanka. The optimum radiation dose for sterilizing male pupae (age 24–48 hours) using a Co 60 source was determined based on the post-irradiation pupal and adult survival in males and induced sterility in females at different doses. Further, the effect of irradiation on mating competitiveness of the selected mosquito strain was assessed under laboratory and semi-field conditions. The optimum release ratio of irradiated males to wild males was assessed in laboratory and semi-field settings.

**Results:**

The optimum radiation dose was 50 Gy among the series of doses (25, 30, 40, 50, 60, and 70 Gy) tested. When pupae were exposed to the optimal radiation dose, 100% pupal survival, 19-day median adult survival time and 99% induced sterility resulted. A 5:1 ratio of irradiated males to non-irradiated laboratory-reared or wild males in laboratory cages resulted in induced sterility of 75% and 62%, respectively. The respective values were 74% and 61% in large semi-field cages. Fried Competitiveness Index (FCI) of irradiated males against wild males of laboratory and wild origin were 0.63 and 0.43 in laboratory cages and 0.57 and 0.55 in large semi-field cages.

**Conclusion:**

The males of *Ae. albopictus* irradiated at 50 Gy are adequately sterile and are competitive against the wild males. The release ratio of 5:1 irradiated males to wild males is a suitable ratio for the field application of SIT. The findings of the study will be important for the development of a protocol for future application of SIT for *Ae. albopictus* in Sri Lanka.

## Introduction

Dengue is a fast-emerging, re-emerging and rapidly spreading mosquito-borne viral disease occurring mainly in the tropics and subtropics from urban to suburban areas. Two mosquito species of the genus *Aedes* are the main vectors of dengue. *Aedes aegypti* (Linnaeus, 1762) originated in Africa and prefers urban habitats, mainly man-made containers. *Aedes albopictus* (Skuse, 1895) originated in Asia and is found in urban to rural habitats breeding peri-domestically. Both species have high vector competence for Dengue Virus (DENV) [1]. A vaccine with high efficacy is not available against dengue infection [2]. Further, there is no specific treatment available for dengue, therefore, controlling vector populations is the most frequent and efficient method for dengue control [3].

In Sri Lanka, dengue is a major public health problem and a leading cause of hospitalization [4]. Sri Lanka has an endemic circulation of all 4 dengue serotypes, but they do not occur in equal abundance [5]. Two main dengue vectors *Ae aegypti* and *Ae*. *albopictus* are present and well established in Sri Lanka [6]. Natural infections of DENV have been reported in both *Ae. aegypti* and *Ae. albopictus* in the country [7,8]. Of the two dengue vector species, *Ae. albopictus* is the most difficult one to control using conventional methods as it prefers to breed in natural habitats [9,10] in addition to artificial containers. Further, resistance to commonly used insecticides has been reported in dengue vectors in Sri Lanka recently [11,12].

Sri Lanka, which has experienced severe dengue epidemics in recent years, has to reconsider its vector control strategies and find suitable solutions to combat the disease. In 2017, 185,688 DF cases were reported in the country, with 45% of the cases being reported from the Western Province [11] where the commercial capital, Colombo is located with the highest population density. Novel control strategies need to be tested, and the feasibility of integrating those with conventional methods in the control of dengue in Sri Lanka has to be investigated.

Radiation-based Sterile Insect Technique (SIT) is a successful vector control technique adopted by several countries to suppress insect pests. The release of sterile mosquitoes using this technique is a potential tool to suppress vector populations [13]. Radiation-based SIT can be integrated with current control methods as an additional tool to suppress the dengue vectors. Knipling was the first to propose releasing sterile males to control natural populations of insect pests using mutagens like gamma radiation in SIT programmes [14]. Over the years, SIT has proven to be a safe, effective and environmentally sound method to suppress, eliminate or contain particular insect pest populations [15]. Successful pilot SIT trials have been carried out on *Ae. albopictus* in Spain [15] and Northern Italy [16,17].

Application of SIT entails the mass production, sterilization and subsequent release of sterile males into a target population in an area-wide and usually integrated pest management strategy. The released sterile males inseminate wild females with sterile sperm. The females subsequently produce unviable offspring, leading to an overall size reduction of the target population. Production of sterile males fit enough to compete with wild males to mate with wild females is a challenge in an SIT programme, and it relies on the proper rearing conditions, use of an optimum radiation dose in retaining physical fitness with adequate survival to induce sterility in the field [18–20].

The objective of this study was to develop protocols of important parameters for future application of SIT for *Ae. albopictus* in Sri Lanka through determining the effective dose of gamma radiation for producing sterile males, assessing the mating competitiveness of irradiated males and determining release ratios of sterile males to wild males of *Ae. albopictus* for the field application of SIT.

## Materials & methods

### Establishment and maintenance of an *Ae. albopictus* colony

A colony of *Ae. albopictus* (Gampaha strain) was established in the laboratory of the Anti-Malaria Campaign, Colombo, Sri Lanka, in 2016 from eggs collected from Kidagammulla, Gampaha (7° 5’ 0“ N, 80° 0’ 30” E), which was the target release area (30 ha) of sterile male mosquitoes. The eggs were collected weekly during a period of one month from the ovitraps placed in the area at a density of 2 traps/ha.. Eggs were hatched using a standard hatching solution (Bacto Nutrient Broth 0.25 g, Brewers’ yeast 0.05 g in 700 ml distilled water) by immersing filter papers containing approximately 5000 eggs overnight. Larvae were reared in plastic trays (Kartell High Impact – 35 cm X 25 cm X 8 cm) at the density of 1 larvae/1 ml in 1000 ml of dechlorinated tap water and fed daily with 4% IAEA diet in solution consisting of 62.5% tuna meal, 25% bovine liver powder, 12.5% Brewer’s yeast, 2 g/L vitamin mixture [21]. The *Ae. albopictus* colony was maintained in plastic cages (30 cm X 30 cm X 30 cm) at a mean temperature of 28 ± 2˚C and a relative humidity of 80% ± 10% with 13-hour light and 11-hour dark with simulated one hour dusk and dawn. Adults were provided with a 10% sucrose solution *ad libitum,* and females were fed with cattle blood once a week using an artificial membrane feeder (5 W1, Hemotek, UK). Plastic cups, half-filled with aged tap water and lined with wet filter paper, were provided for egg laying. The papers with eggs were collected, dried slowly and stored in zip-lock bags for several weeks. Eggs were hatched and reared as described whenever pupae were required for experiments. Larvae, pupae and adults of F1-F15 were used for the experiments during the period from 2016 to 2019.

#### Radiation facility.

The irradiator used for the study was a Gamma cell (Gamma 220, Atomic Energy of Canada Ltd., Co60) which can give small doses (<100 Gy) required for mosquito irradiation and is located at the Human Tissue Bank, Colombo. The exposure time was calculated based on the dose mapping done for this irradiator before the experiments described in this paper were started. The dose rate of the irradiator was 1.22 kGy/hour on 2015-04-21.

### Determination of the optimum radiation dose for sterilizing male pupae

The minimum exposure dose needed to achieve more than 99% sterility in male *Aedes albopictus* was determined using a dose-response curve. The chosen dose was based on exponential growth ranging from 25 Gy to 70 Gy. Approximately one hundred males of pupae were exposed to each dose.

Pupae of *Ae. albopictus* (Gampaha strain) reared in the insectary were separated into males and females using a stereo microscope (10 X 4) by checking their terminalia. The male pupae were transported in plastic cups containing water to the irradiation facility. Batches of male pupae (n = 32) aged 24–48 hours from the F1 generation were inserted in plastic vials (height 7 cm, diameter 3.5 cm) with a small amount of water (10 ml) and irradiated with gamma-rays with doses of 25, 30, 40, 50, 60 and 70 Gy. As a control of fertility, we kept a non-irradiated group. Three replicates of 32 pupae per vial were irradiated separately for each dose. The experiment was repeated for pupae of the F8 generation. All the replicates for F1 and F8 generations were pooled for the analysis. In addition to the male pupae, 2 replicates of female pupae (n = 50) of the same cohort were irradiated under the same conditions at 50 Gy. At each time of irradiation, Fricke dosimetry [22] was performed to measure the actual radiation dose received.

#### Pupal mortality, adult survival, fecundity and induced sterility after irradiation.

Irradiated male pupae were transferred to plastic cages (30 cm X 30 cm X 30 cm) in plastic cups containing water for emergence and supplied with 10% sucrose solution with continuous access. The post-irradiation pupal mortality rate was recorded after 48 hours for each radiation dose. Females pupae from the same cohort (F1) were also obtained. After emergence, virgin females in equal numbers to the males were introduced into each cage and allowed to mate with continuous access to a 10% sugar solution. Mortality of adult males was recorded daily for a month. After 5 days, females were fed with cattle blood and engorged females were isolated just after blood feeding into individual plastic vials (7 cm height and 3.5 cm diameter) lined with wet filter paper and with 10 ml of water. After 5 days, females were removed, and the filter papers with eggs were allowed to dry slowly for one week inside the same vial. The number of eggs in the dried filter paper laid by a single female (fecundity) was enumerated under a stereo microscope. Filter papers with eggs were then returned to individual tubes and filled with hatching solution. After 12 hours, the number of L1 was recorded and hatch rates were calculated for individual females. To assess the insemination rate of female cages with irradiated males, the spermathecae of females were dissected under a stereo microscope (2 X 10) and the presence of spermatozoa was observed under a light microscope at (10 X 40) magnification.

Irradiated female pupae were transferred into a cage in a plastic cup, and after emergence, non-irradiated males of the same cohort were introduced in equal numbers (n = 50). The same procedures of feeding and egg laying described above were followed and checked for eggs in individual females.

### Mating competitiveness and optimum release ratios of sterile males under laboratory conditions

#### Irradiation of laboratory-reared male pupae.

From the previous experiment, the dose of 50 Gy was selected as the sterilizing dose for the studies on male mating competitiveness and optimum release ratio using batches of *Ae. albopictus* pupae, Gampaha strain (F8, 28–40 hours, n = 350).

#### Irradiated males with untreated laboratory-reared males to mate with laboratory-reared female mosquitoes.

Three possible sterile-to-fertile male ratios increasing the proportion of irradiated males were tested for mating competitiveness in laboratory cages (30 cm X 30 cm X 30 cm). After 3 days of emergence, irradiated and untreated males were released into the cages to compete with each other to find female mates. Females of the same cohort were introduced after all the males were released. The ratios were 1:1:1, 3:1:1 and 5:1:1 in which they were sterile males: fertile males: virgin females. The number of virgin females was kept at 100 in all the cages. A fertile control was set with untreated males and 100 virgin females. Sterile control was set with irradiated males and 100 virgin females. Mosquitoes were allowed to mate with access to a 10% sucrose solution for 3 days. After 3 days, blood-feeding was carried out 3 times for 2 days. Eggs were collected and counted. The hatch rates were calculated for individual females as in the previous experiments.

#### Irradiated males with wild males to mate with wild female mosquitoes.

A similar experiment was conducted for irradiated males, replacing laboratory-reared fertile males and virgin females with wild males and wild females. Wild fertile males and females reared from field-collected eggs from Gampaha (F1) were used to compete and mate with laboratory-reared irradiated males in the same ratios used in the previous experiment. Fertile control was set with wild males and wild virgin females 1:1, and sterile control was set with irradiated males with wild virgin females 1:1. Male and female pupae used for the competitiveness assessment were separated by the mechanical sieving method.

### Mating competitiveness and optimum release ratio of sterile males under semi-field conditions

The experiment was performed in semi-field cages following the same experimental ratios as described under the laboratory conditions.

**Fig 1:**
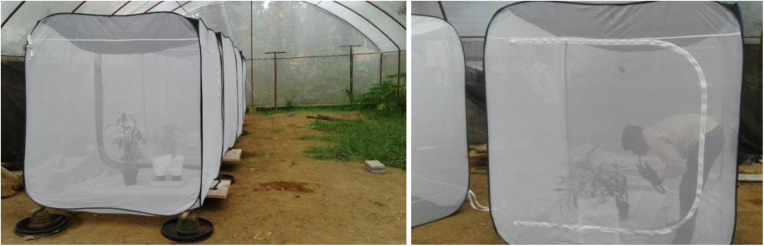
Experimental semi-field cages (1.82 m X 1.21 m X 1.21 m).

The irradiated male, untreated male and female pupae were kept separately in laboratory cages (30 cm X 30 cm X 30 cm) until emergence with access to 10% sugar solution in the insectary. Three days after emergence, the cages were taken in an air-conditioned vehicle to the field area where semi-field cages (1.82 m X 1.21 m X 1.21 m) were set up inside a greenhouse in Gampaha district (Fig 1), about 25 km away from the laboratory. This work was performed following the basic idea of semi-field conditions from previous studies [23] and adapting to the objectives of current study and local conditions.

Five semi-field cages were set up inside the green house on ant traps. Inside the cages 10% sugar solutions were placed with immersed filter papers on ant traps. A small potted plant and a wet cardboard box were also placed in each cage to provide resting sites. In semi-field cages. During the experiment, the temperature in the greenhouse fluctuated between 30–37°C. Relative humidity was approximately 60% during the daytime and the work was carried out under ambient light.

### Irradiated males with untreated laboratory-reared males to mate with laboratory-reared female mosquitoes

The initial competitiveness experiment under semi-field conditions was carried out using irradiated males and untreated laboratory-reared males to mate with laboratory-reared female mosquitoes. Mosquitoes brought in laboratory cages were released into semi-field cages following all four ratios used in the previous experiment. Female mosquitoes were released in to each cage as the end to give equal opportunity to fertile and sterile males for mating. Mosquitoes were allowed to mate with access to 10% sucrose solution. After 3 days, females were collected using mouth aspirators into five small cages (30 cm X 30 cm X 30 cm) and brought to the insectary. Blood feeding, tubing egging and hatching were done as described in the previous section on mating under laboratory conditions.

### Irradiated males with wild males to mate with wild female mosquitoes

The following competitiveness experiment under semi-field conditions was performed using wild males and wild females replacing laboratory-reared fertile males and females respectively. Wild fertile males and females were obtained by rearing field-collected eggs (F0) from the Gampaha study area. In this experiment, wild males were allowed to compete with laboratory-reared (F39) irradiated males to mate with wild females. this is the situation expected to occur when irradiated males are released into the wild *Ae. albopictus* population. Sterile males were released in different ratios into semi-field cages which were set up inside the greenhouse (Fig 1). Eggs were obtained and hatched as mentioned in the previous experiment.

### Ethical statement

Ethical clearance was obtained from the Ethical Review committees of the Faculty of Medicine, University of Kelaniya (P/239/12/2014) and Institute of Biology, Colombo 07, Sri Lanka (ERC IOBSL 207 02 2020). Work permits were not required as the study areas were not declared protected areas.Informed written consent was taken from an adult member of the household for sampling mosquitoes.

### Data analysis

Data were analysed to determine the optimum dose of radiation to sterilize pupae. The Kaplan-Meier survival analysis was used to estimate the median survival times of irradiated adult males held in cages. The Log-Rank test was used to perform pairwise comparisons of survival curves from the different doses at the level of significance α < 0.05. Post-irradiation pupal mortality rate and insemination rate were calculated for each irradiation dose. The number of eggs laid and egg hatch rate were calculated for individual females and mean values were taken for each replicate of different doses. Induced sterility was calculated as described by Yamada *et al.* [24]. General Linear Models (GLM) followed by Tukey’s Honest Significant Difference (HSD) were used to observe differences in pupal mortality, insemination rate, mean number of eggs per female, fertility/egg hatch rate and induced sterility in response to different irradiation doses.

To assess the mating competitiveness and optimum release ratios, the variance in egg hatch rates and induced sterility of irradiated males among the 3 combination ratios was tested (ANOVA, Post Hoc) for laboratory cages and semi-field cages. Fried competitive index ‘FCI’ was calculated for irradiated and laboratory males, irradiated and wild males for laboratory cages and semi-field cages using hatch rates of the fertile control, sterile control and 1:1:1 experimental cage [25,26]. FCI = {(Hn – Ho)/ Ho – Hs} x N/S where Hn = egg hatch rate of females mated with untreated and sterile males, Hs = egg hatch rate of females mated with sterile males, Ho = observed egg hatch rate, N = number of untreated males and S = number of sterile males. The FCI was compared between laboratory bread and wild males with irradiated males and under laboratory and semi-field conditions. The data analysis was performed using SPSS version 20.

## Results

### Determination of the optimum radiation dose for sterilization of *Ae. albopictus*

#### Dosimetry.

Frickey Dosimetry results confirmed that all doses of gamma rays were delivered within the target dose ± 3 Gy during the exposure at different doses.

#### Pupal mortality.

**Fig 2:**
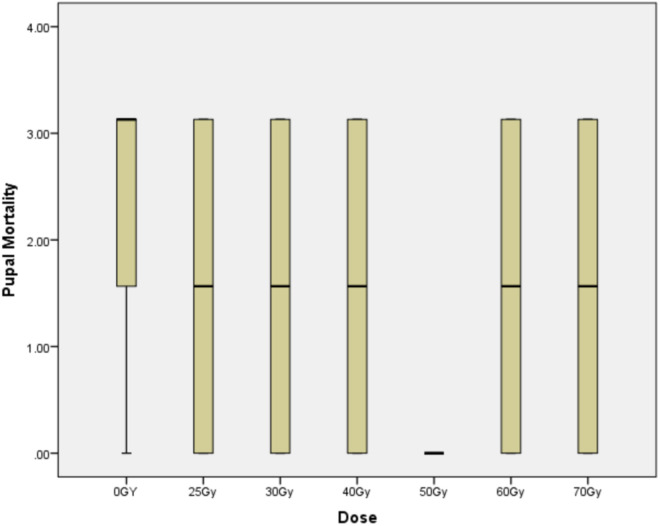
Box-plot of pupal mortality at different irradiation doses.

Mortality of male pupae after radiation exposure ranged from 0 to 2.08 ± 1.04% among the different radiation doses and was not significantly different among radiation treatments and from the control (F = 0.239, P = 0.916) (Fig 2).

#### Adult survival.

**Fig 3:**
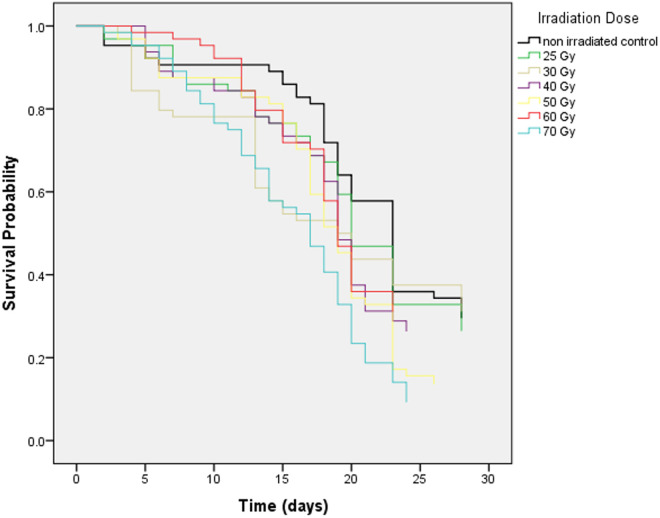
Survival curves of *Ae. albopictus* males at different irradiation doses.

The adult male survival rates were significantly influenced by the radiation dose (χ2 = 23.86, df = 6, p < 0.001, Fig 3). The survival of non-irradiated males was similar to that of males irradiated with a dose of 30 Gy or lower (i.e., 30 and 25 Gy; p < 0.45) but significantly higher than the survival of males irradiated with a dose greater than 30 Gy (i.e., 40, 50 and 70 Gy; p < 0.04). The lower survival rate was observed with the highest irradiation dose used of 70 Gy. The median survival times showed a decreasing trend with the increase of the radiation dose (Table 1).

**Table 1:** Median survival times of irradiated males at different doses. (Letters a,b,c represent homogenous groups).

Dose	Median survival time (No. of days ± SE)
Control	23 ± 0.823^a^
25 Gy	20 ± 0.939^a^
30 Gy	20 ± 2.778^a^
40 Gy	19 ± 0.5^b^
50 Gy	19 ± 0.747^b^
60 Gy	19 ± 0.552^b^
70 Gy	17 ± 1.198^b^

#### Insemination rate and fecundity.

**Fig 4:**
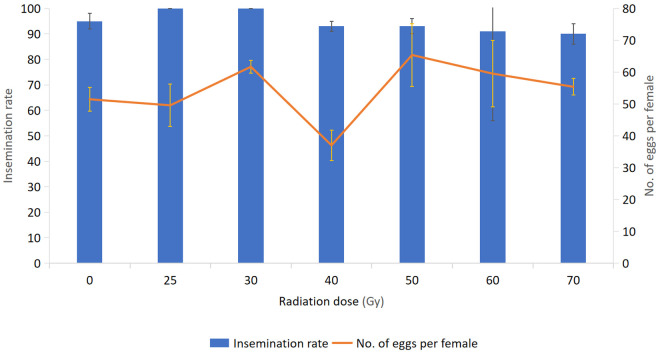
Insemination rate and fecundity of *Ae. albopictus* females mated with males irradiated at different doses.

The insemination rate and fecundity (the number of eggs laid after the first blood meal) of *Ae. albopictus* at different irradiation doses are given in Fig 4. The insemination rate of females mated with males irradiated at different doses varied between 95% ± 3 (mean ± SE) and 100% and was not significantly different among tested doses (p = 0.510) and all were above 90% including control (F1 generation). Fecundity ranged from 37.025 ± 4.72 eggs per female to 65.37 ± 9.87 eggs per female. However, this parameter did not differ significantly among the different radiation doses tested and from the control (F = 2.19, p = 0.110).

#### Egg hatch rate and induced sterility.

**Fig 5:**
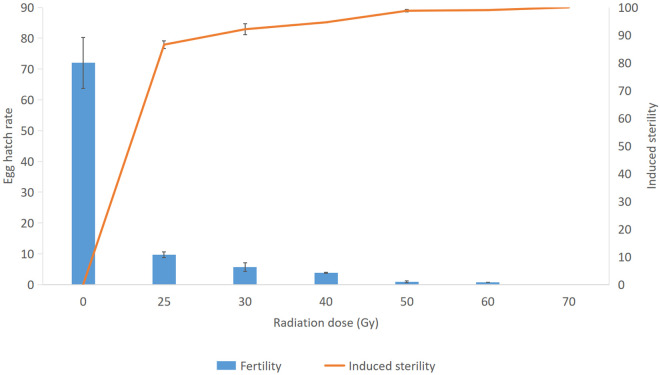
Egg hatch rate and induced sterility of *Ae.a.* *lbopictus* females mated with males irradiated with different doses.

The egg hatch rate resulting from mating with irradiated males significantly differed from the non-irradiated control (p = 0.00002) and decreased with increasing radiation dose and became 0 at 70 Gy. Induced sterility showed significant differences among doses of 40, 50 and 60 Gy. The mean induced sterility increased with the radiation dose and was significantly different among doses. It was approximately 99% at the doses of 50 and 60 Gy (Fig 5).

The radiation doses 50 Gy and 60 Gy gave the highest induced sterility which was approximately 99% and the irradiated males had 19 days of median survival time. Based on these results, 50 Gy was selected as the lowest dose giving adequate sterilization in *Ae. albopictus* (Gampaha strain) for competitiveness studies (Fig 5).

Adult females emerged from the pupae of *Ae. albopictus* (Gampaha strain) exposed to radiation (n = 100, 50 Gy) and mated with non-irradiated males did not lay any eggs (Table 2).

**Table 2:** Effect of radiation dose on insemination rate, fecundity, egg hatch rate and induced sterility (n = 4).

Radiation dose (Gy)	Proportion of insemination ± SEM	No. of eggs per female ± SEM	Egg hatch rate % ± SEM	Induced sterility % ± SEM
0	0.95 ± 0.03	51.47 ± 3.8	71.96 ± 8.22^a^	0^a^
25	1.00	49.58 ± 6.67	9.68 ± 0.99^b^	86.56 ± 1.38^b^
30	1.00	61.68 ± 2.02	5.68 ± 1.44^b^	92.11 ± 2.00^c^
40	0.93 ± 0.02	37.025 ± 4.72	3.88 ± 0.16^b^	94.61 ± 0.23 ^c,d^
50	0.93 ± 0.03	65.37 ± 9.87	0.88 ± 0.34^b^	98.78 ± 0.47 ^d,e^
60	0.91 ± 0.35	59.52 ± 10.39	0.71 ± 0.06^b^	99.02 ± 0.08 ^d,e^
70	0.90 ± 0.04	55.39 ± 2.61	0	100^e^

### Mating competitiveness and release ratios of irradiated males under laboratory and semi-field conditions

Results of mating competitiveness tests are given in Table 3.

**Table 3:** Hatch rate and induced sterility at different combinations in laboratory cages.

Experiment	Ratio	No. of eggs per female ± SEM	Hatch rate % ± SEM	Induced sterility % ± SEM
Irradiated male (Lab): Nonirradiated male (Lab): Female (Lab)	0:1:1 (Fertile control)	36.24 ± 4.62^a^	80.10 ± 0.58 ^a^	0 ^a^
	1:0:1(Sterile control)	37.12 ± 2.76 ^a^	4.92 ± 0.32 ^b^	93.85 ± 0.40 ^b^
	1:1:1	40.17 ± 1.81 ^a,b^	51.12 ± 2.71 ^c^	36.18 ± 3.38 ^c^
	3:1:1	39.37 ± 8.25 ^a,b^	35.70 ± 3.43 ^d^	55.43 ± 4.28 ^d^
	5:1:1	37.94 ± 1.56 ^a^	19.84 ± 0.08^e^	75.22 ± 0.09 ^e^
Irradiated male (Lab): Non-irradiated male (Wild): Female (Wild))	0:1:1(Fertile control)	67.93 ± 4.51 ^a,b^	81.19 ± 0.95 ^a^	0.00^a^
	1:0:1(Sterile control)	77.12 ± 1.28 ^c^	2.67 ± 0.2 ^b^	96.74 ± 0.24 ^b^
	1:1:1	75.94 ± 6.05^b^	57.43 ± 0.2 ^c^	29.80± 0.18^c^
	3:1:1	62.91 ± 14.48 ^a,b^	33.97 ± 3.37 ^d^	58.48 ± 4.11^d^
	5:1:1	64.79 ± 8.56 ^a,b^	31.32 ± 2.43 ^d^	61.72 ± 2.97^d^

#### Under laboratory conditions.

In the first experiment, when fertile males and females were of laboratory origin, the number of eggs produced per female was not significantly different between different combinations of irradiated males (Table 3). The egg hatch rate decreased from 51% to 36% and then to 20% when the proportion of irradiated males increased from 1:1–2:1and 3:1 respectively and the induced sterility increased correspondingly reaching 75% when irradiated males were present 5 times. Egg hatch rates were significantly different among different combinations and from the fertile control (p < 0.05). Further, the differences between mean hatch rates of ratios 3:1:1 and 5:1:1 were statistically significant. The induced sterility also differed among tested combinations with statistical significance.

When irradiated laboratory males were caged with wild fertile males and wild females in the same ratios as in the laboratory cages, the egg hatch rates decreased with the increase of the proportion of irradiated males. However, in combinations with 3:1 and 5:1 of irradiated males egg hatch rates and induced sterility were not significantly different between the 2 combinations. Further, a slightly less induced sterility was seen at 1:1:1 and 5:1:1 ratios compared to previous experiments with laboratory fertile males.

#### Under semi-field conditions.

The results in terms of number of eggs per female, egg hatch rate and induced sterility are shown in Table 4. The number of eggs per female remained with no statistically significant differences among different ratios. Egg hatch rate in tested combinations decreased with the increasing proportion of irradiated males in large cages reaching 20% with laboratory males and 31% with wild males at 5:1:1 combination. When competed with non-irradiated wild males the hatch rates of combinations at 3:1 and 5:1 of sterile males were not significantly different from each other (p = 0.731, F = 130.49). Induced sterility increased correspondingly with the increase of irradiated sterile male ratio ranging from 34% at the ratio of 1:1:1–74% at 5:1:1 when competed with laboratory-reared non-irradiated males. However, with wild non-irradiated males, the induced sterility was slightly less but not different statistically (p = 0.615, F = 460.28). Induced sterility between the 2 combinations with 3X and 5X irradiated males with wild males was not significantly different from each other.

**Table 4:** Egg hatch rates and induced sterility at different combinations at semi-field cages.

Experiment	Ratio	No. of eggs per female ± SEM	Egg hatch rate % ± SEM	Induced sterility % ± SEM
Irradiated male (Lab): Non-irradiated male (Lab): Female (Lab)	0:1:1 (Fertile control)	44.40 ± 14.20	80.49 ± 3.75 ^a^	0.00 ^a^
	1:0:1 (Sterile control)	52.91 ± 26.22	3.62 ± 1.16 ^b^	95.51 ± 1.45^e^
	1:1:1	46.25 ± 0.69	52.54 ± 1.34^e^	34.73 ± 1.70^b^
	3:1:1	40.39 ± 9.71	33.17 ± 2.53 ^c d^	58.80 ± 3.14^c, d^
	5:1:1	49 ± 8.85	20.47 ± 2.47 ^c^	74.56 ± 3.07 ^d^
Irradiated male (Lab): Non-irradiated male (Wild): Female (Wild)	0:1:1	67.12 ± 3.30	81.97 ± 2.31 ^a^	0.00 ^a^
	1:0:1	61.16 ± 0.001	3.37 ± 0.30 ^b^	95.88 ± 0.37^e^
	1:1:1	56.45 ± 13.83	54.15 ± 2.94^e^	33.94 ± 3.59^b^
	3:1:1	62.53 ± 4.86	39.08 ± 3.50 ^d `e^	52.32 ± 4.26^c^
	5:1:1	64.46 ± 0.94	31.74 ± 4.76 ^c d^	61.28 ± 5.80 ^c, d^

The egg hatch rates and induced sterility showed a more or less similar trend in both laboratory and semi-field conditions for 3 different ratios of sterile males when competed with fertile males irrespective of their origin (laboratory or wild), though wild females had given higher number of eggs compared to laboratory-reared females in both laboratory and semi-field conditions. The lowest hatch rate and the highest induced sterility resulted when sterile males and fertile males were 5:1 in all 4 experiments.

### Fried competitiveness index (FCI)

Fried Competitiveness Index (FCI) was calculated for the ratio of 1:1:1 of Irradiated male: Non-irradiated male: Female in laboratory small cages and semi-field large cages (Table 5).

**Table 5:** Fried competitiveness index (FCI).

Setting	Group	Ratio	Competitiveness Index (Mean±SD)
Laboratory (Small cage)	Irradiated male (Lab): Non-irradiated male (Lab): Female (Lab)	1:1:1	0.63 ± 0.14
	Irradiated male (Lab): Non-irradiated male (Wild): Female (Wild)	1:1:1	0.43 ± 0.03
Semi-field (Large cage)	Irradiated male (Lab): Non-irradiated male (Lab): Female (Lab)	1:1:1	0.57 ± 0.39
	Irradiated male (Lab): Non-irradiated male (Wild): Female (Wild)	1:1:1	0.55 ± 0.06

In the laboratory settings, the FCI was 0.63 and closer to the standard 0.75 [27] when irradiated males competed with laboratory males. When the competition was with wild males the FCI became lower (0.43) than the above, however, irradiated males were nearly as competitive as half as wild males though the value is low compared to the standards. In semi-field settings, the values of FCI are almost equal and around 0.5 regardless of the origin of untreated males indicating irradiated males are as competitive as half as untreated laboratory-reared males or wild males of *Ae. Albopictus.*

## Discussion

In this study, we conducted laboratory and semi-field experiments to determine the optimal levels of some key parameters for SIT in order to develop a protocol for applying radiation-based SIT to control *Ae. albopictus* in Sri Lanka.

Choosing the right radiation dose is crucial for a successful SIT program. The optimization of this dose requires precise dosimetry to ensure the insect becomes sterile while retaining competitiveness for successful mating [20,28]. According to the guidelines from WHO and IAEA [27], the suitable radiation dose for male *Ae. albopictus* should result in 99–100% sterility while maintaining an adequate survival rate and mating ability. Therefore, the radiation dose that achieves 96–98% sterility with a satisfactory survival rate and mating ability for male *Ae. albopictus* was selected.

Researchers have investigated the effects of radiation doses on the pupal mortality, adult survival and induced sterility of *Ae. albopictus* males, as well as the key factors influencing these responses [24,29]. In this study, it was found that pupal survival was not significantly affected by radiation up to 70 Gy. In previous studies, it was observed that exposure to radiation doses of 30–35 Gy did not have a negative impact on the lifespan of *Ae. albopictus* [21]. The research indicated that the survival of adult males remained unaffected even when subjected to doses up to 40 Gy after 30 hours of the pupal stage. Similarly, other studies [26,29] also found no significant difference in survival between control groups and those exposed to doses up to 50 Gy for the same species. In the current study, it was noted that pupae exposed to radiation up to 70 Gy exhibited lower mortality rates and successful emergence, possibly due to being placed in a small amount of water during radiation, creating a low-oxygen environment. Research has shown that *Ae. aegypti* pupae exposed to severe hypoxic conditions for less than 1 hour before and during radiation exhibited no differences in mortality compared to the control group at doses up to 50 Gy [30]. According to a previous study acceptable levels of pupal mortality and adult survival in laboratory conditions are defined as <10% instantaneous male mortality and <10% reduction in survival times at the target radiation dose [27]. In our study, both these parameters were in the expected levels. However, more sample number and more replicates are necessary to confirm.

It was found that the insemination rate of the irradiated males among different doses and non-irradiated controls was above 90% based on spermatheca dissection of blood-fed females one week after feeding. The ability of irradiated males to inseminate females has also been studied for *Aedes* [23,31]. No difference in insemination rate has been seen in response to used radiation doses in *Ae. albopictus*. This evidence shows that the irradiation at this selected dose range does not adversely affect the mating activity of males which was further evident in personal observations of mating pairs of irradiated males with non-irradiated females in laboratory and semi-field cages during the study.

Studies have been done on the effects of radiation on adult survival and the mating competitiveness of *Ae. albopictus* using gamma rays [21,23,26,29] and using X-rays [32]. The optimal radiation dose for mosquito sterilization has been researched in SIT for different species as well. For *An. arabiensis* SIT trials in Sudan, 70 Gy was shown to be a partially sterilizing dose [33]. For *An. coluzzii,* 90 Gy has been used for mating competitiveness studies [34]. The most effective sterilizing dose that did not adversely affect the male of *Ae, aegypti* was determined to be 50–55 Gy [35,36]. For *Ae. albopictus* 80 Gy has been used from a Co 60 source as the minimum acceptable dose for sterilizing pupae in water in initial research work [37] but reduced up to 30 Gy in later work [16]. Balestrino *et al*. [21] have used 35 Gy for pupae irradiation from a Cs 137 source for which in the present study 99% sterility was not obtained. In the present study, Kaplan-Meier survival analysis has shown 50 Gy as the optimal radiation dose giving required induced sterility in male pupae of *Ae. albopictus.* Here, pupal irradiation was carried out when pupae were kept in 20 ml of water in plastic tubes for 1–2 hours during travel to the irradiator. This may cause hypoxic conditions in the water due to the respiration of pupae. This has an attenuation effect on irradiation and may result in increased hatch rates where a 3.8-fold increase in residual fertility had been seen in hypoxic versus normoxic conditions in *Ae. albopictus* at 35 Gy [24].

Remaining residual fertility at 50 Gy in this study was 2–4%. However, due to good pupal survival, emergence and adult longevity, 50 Gy was selected as the optimum dose. Complete female sterility at the selected dose was important as in accidental female release the ability to transmission of the dengue is equal to the non-irradiated females [38]. At the dose of 50 Gy the females did not lay eggs and were fully sterile.

Mating competition of sterile males [27], FCI is expected to be > 0.7 in laboratory testing and >0.5 under semi-field conditions. The current study resulted in a very close value, 0.63 ± 0.14 of FCI in the laboratory testing with equivalent non-irradiated laboratory counterparts, but when competed with wild counterparts, the competitiveness was slightly lower (0.43 ± 0.03). The reduction of sterile male competitiveness may be due to the exposure to artificial conditions in the laboratory of the fertile males of *Ae. Albopictus* compared to wild males. In semi-field testing the competitiveness is above the expected value.

In SIT simulation experiments, a 5:1 sterile-to-wild male ratio allowed a 2-fold reduction of the wild population’s fertility [23]. In the present study, with the increased ratios of sterile males, the egg hatch rates decreased. Even though >90% reduction in viable egg production in mosquito populations is expected in semi-field studies for an over-flooding ratio of 10:1, this study tested up to a 5:1 ratio and got a reduction of 60% of viable egg production.

## Conclusion

The results suggest that male *Ae. albopictus* mosquitoes irradiated at 50 Gy can effectively induce sterility in females. A release ratio of 5:1 irradiated males to wild males resulted in significantly lower hatch rates, making it a promising ratio for implementing the Sterile Insect Technique (SIT). These findings could serve as a foundation for establishing a standardized SIT protocol to control *Ae. albopictus* populations in Sri Lanka.

## Supporting information

S1 Text **S1 File**. Adult survival output. **S2 File**. Dose Mapping output. **S3 File**. Mating competitiveness data set. **S4 File**. Dose mapping of Gamma Cell.
(ZIP)

## Abbreviations

SIT: Sterile Insect Technique
FCI: Fried Competitive Index
DENV: Dengue Virus.
